# Rapid and Easy-to-Use Method for Accurate Characterization of Target Binding and Kinetics of Magnetic Particle Bioconjugates for Biosensing

**DOI:** 10.3390/s21082802

**Published:** 2021-04-15

**Authors:** Averyan V. Pushkarev, Alexey V. Orlov, Sergey L. Znoyko, Vera A. Bragina, Petr I. Nikitin

**Affiliations:** 1 Prokhorov General Physics Institute of the Russian Academy of Sciences, 38 Vavilov Str., 119991 Moscow, Russia; pushkarev@phystech.edu (A.V.P.); alexey.orlov@kapella.gpi.ru (A.V.O.); znoykosl@nsc.gpi.ru (S.L.Z.); bragina_vera@nsc.gpi.ru (V.A.B.); 2 Moscow Institute of Physics and Technology, 9 Institutskii per., Dolgoprudny, 141700 Moscow Region, Russia

**Keywords:** magnetic sensors, particle targeting, immunosensing of cardiac markers, biosensors for staphylococcal enterotoxins detection, lateral flow sensors

## Abstract

The ever-increasing use of magnetic particle bioconjugates (MPB) in biosensors calls for methods of comprehensive characterization of their interaction with targets. Label-free optical sensors commonly used for studying inter-molecular interactions have limited potential for MPB because of their large size and multi-component non-transparent structure. We present an easy-to-use method that requires only three 20-min express measurements to determine the key parameters for selection of optimal MPB for a biosensor: kinetic and equilibrium characteristics, and a fraction of biomolecules on the MPB surface that are capable of active targeting. The method also provides a prognostic dependence of MPB targeting efficiency upon interaction duration and sample volume. These features are possible due to joining a magnetic lateral flow assay, a highly sensitive sensor for MPB detection by the magnetic particle quantification technique, and a novel mathematical model that explicitly describes the MPB-target interactions and does not comprise parameters to be fitted additionally. The method was demonstrated by experiments on MPB targeting of cardiac troponin I and staphylococcal enterotoxin B. The validation by an independent label-free technique of spectral-correlation interferometry showed good correlation between the results obtained by both methods. The presented method can be applied to other targets for faster development and selection of MPB for affinity sensors, analytical technologies, and realization of novel concepts of MPB-based biosensing in vivo.

## 1. Introduction

Novel sensing systems increasingly use materials and structures based on magnetic particle bioconjugates (MPB) due to their unique properties [1,2,3,4,5,6,7]. Indeed, MPB as carriers, which can be manipulated by an external magnetic field, have revolutionized approaches to development of biosensors and biomedical research [8,9,10]. In addition, using MPB as labels detectable by magnetic sensors is among the most promising strategies to enhance the rapidity and sensitivity of immunoanalytical methods for medical diagnostics, food control, biosafety, etc. [11,12,13,14,15,16,17]. Moreover, recent developments have brought fundamentally new MPB-based concepts for drug delivery and biosensing in vivo, which employ the fact that the local concentration of targets in the area of interest may considerably exceed their average concentration in blood [18,19,20]. In these advanced concepts, initiating therapeutic and/or diagnostic actions of the MPB structures is presumed solely in the areas of elevated concentrations of specific markers. All the mentioned factors call for methods of accurate measuring the target-binding characteristics, kinetics, and affinity of MPB.

To date, the most accurate and efficient tools for investigation of inter-molecular interactions and measuring their kinetic characteristics have been direct label-free optical sensors [21,22,23,24,25]. These sensors employ diverse detection principles including surface plasmon resonance [26,27,28,29,30]; slot-waveguide, ring, and disk resonators [31,32,33]; interferometry [34,35,36]; bio-photonic sensing cells [37,38], acoustic and graphene-based sensors [39,40], etc. The label-free sensors register in real time the binding of biorecognition molecules with target molecules immobilized on a sensor chip.

Despite remarkable achievements of the label-free sensors in the studies of inter-molecular interactions such as those of antibody–antigen, ligand–receptor, lectin–carbohydrate, etc., their applicability for exploration of MPB–target interactions is confined to a few types of MPB [41,42,43]. That is due to much larger size of MPB compared to that of a molecule. In addition, direct investigation of the multi-component opaque MPB structure by the label-free sensors has been greatly hampered. Moreover, virtually all mentioned methods permit studying the interactions solely with target molecules adsorbed on a solid surface of a sensor chip. The sorption may change the molecule conformation so that the recognizing areas of molecules targeted by MPB may be hindered. These factors may compromise the measured parameters [26,27,44]. 

Hence, two major challenges for the methods of measuring the kinetic characteristics of MPB are as follows: (i) investigation of entire MPB rather than its biorecognition components; (ii) study of MPB interaction with free, non-sorbed targets.

Here, we present a novel method that meets both mentioned challenges and permits accurate determination of target-binding characteristics and kinetics of MPB using as little as three rapid measurements. The method is based on the following centerpieces: (i) an original experimental setup that implements an express magnetic lateral flow (LF) assay with a highly sensitive sensor for MPB detection by the magnetic particle quantification (MPQ) technique; and (ii) a mathematical model that explicitly describes formation and evolution of MPB-target complexes and does not comprise parameters to be fitted additionally. The fast, accurate and convenient procedures for obtaining both kinetic and equilibrium constants, as well as for a prognosis of interaction dynamics have been demonstrated with MPB targeting of two non-sorbed protein macromolecules: a cardiac marker of cardiac troponin I (cTnI) and a bacterial toxin of staphylococcal enterotoxin B (SEB). The obtained characteristics have been validated with an independent label-free method of spectral-correlation interferometry, which showed good correlation of the results.

## 2. Materials and Methods

### 2.1. Reagents

The following reagents were used: mouse monoclonal antibody against cardiac troponin I, 19C7 and 16A11 clones, and troponin I-T-C complex (HyTest Ltd., Moscow, Russia); mouse monoclonal antibody against staphylococcal enterotoxin B, clones S643 and S222; SEB antigen (RCMDT, Moscow, Russia); bicinchoninic acid (BCA) Protein Assay Kit (Thermo Fisher Scientific, Waltham, MA, USA); (3-Aminopropyl)triethoxysilane (APTES); N-(3-Dimethylaminopropyl)-N-ethylcarbodiimide hydrochloride (EDC); Tween-20; bovine serum albumin (BSA); N-hydroxysulfosuccinimide sodium salt (sulfo-NHS); succinic anhydride (Sigma Aldrich, Saint Louis, MO, USA); 2-(N-morpholino) ethanesulfonic acid (MES) (AppliChem, Darmstadt, Germany); sulfuric acid; methanol; dimethylformamide (Chimmed, Moscow, Russia). Superparamagnetic carboxyl-modified (COOH−) particles of ≈200 nm in diameter were purchased from Estapor—Merck Millipore, France (listed as “Bio-Estapor Microspheres” in the manufacturer’s catalogue). The exact particle size is indicated below according to the manufacturer documents. Sample pads and absorbent sinks/wicking pads were from Ahlstrom CytoSep, Finland; backing cards—from Lohmann, San Jose, CA, USA; nitrocellulose membrane UniSart CN95—from Sartorius Stedim Biotech GmbH, Goettingen, Germany.

### 2.2. Preparation of Magnetic Particle Bioconjugates

The magnetic particle bioconjugates were prepared by a carbodiimide method of covalent immobilization of monoclonal antibodies on carboxyl-modified magnetic particles [45,46,47]. Briefly, 1 mg of the particles was twice magnetically washed with MES buffer (0.1 M, pH 5.0). The particles were exposed to a solution of 6 mg EDC and 3 mg of sulfo-NHS in 50 μL of MES buffer. Then 50 μg of monoclonal antibody in borate buffer (0.1 M, pH 8.6) was added followed by 2-h incubation. After that, 10 μL of 10% BSA was added and incubated for 1 h to ensure colloidal stability of the particles and blocking the carbodiimide-activated COOH-groups on their surface that did not reacted with antibody. Finally, the conjugates were thrice magnetically washed with PBS buffer (10 mM, pH 7.4). The magnetic washing procedure consisted in the particle collection with a magnetic separation rack followed by removing and discarding the supernatant. The used carbodiimide EDC/NHS method is among the major techniques for immobilization of monoclonal antibody onto the surface of magnetic nanoparticles. The method, though, does not allow proper orientation of all antibodies, and some of them may bind to nanoparticles by their Fab fragments.

### 2.3. Technique of Magnetic Particle Quantification (MPQ)

The MPQ registration technique pioneered by Nikitin et al. [48] is intended for 3D quantification of superparamagnetic particles by non-linear magnetization with ultrahigh sensitivity up to 60 zM [49]. The technique principle can be briefly described as follows: the particles are subjected to a two-frequency alternating magnetic field, and their non-linear response is recorded at frequencies, which are a linear combination of the excitation frequencies or harmonics of one excitation frequency [48]. The MPQ can register magnetic materials over the entire volume of a sample regardless of its optical transparency. New MPQ readers were fabricated for this research that implemented registration within 10 s on average and offered a wide 7-order linear dynamic range. A detailed description of the MPQ method, its scheme, characterization, and applications were reported earlier [7,10,48,49].

### 2.4. Fabrication of Lateral Flow Strips and LF Assay Procedure

The proposed method as a tool for investigation of target recognition by magnetic nanobioconjugates is based on the rapid lateral flow format. The entire measuring procedure, including the lateral flow test, particle detection and data processing, takes 20 min. This tool is applied to investigation of MPB targeting. The targeting process can last any time on demand, e.g., we demonstrate how the method works for targeting durations from 30 min to 24 h before measurements. Importantly, each measurement still takes 20 min. 

The LF test is realized in the sandwich format [50,51] with MPB as labels detected by an MPQ sensor. To prepare an LF strip, a sample pad, nitrocellulose membrane and absorbent pad were assembled on an adhesive backing card with overlapping. The test line (TL) was formed by deposition of a solution of monoclonal antibody against either cTnI or SEB onto the nitrocellulose membrane at a jetting rate of 1 µL/cm. The fully assembled card was dried for 4 h at +37 °C and then cut into 3-mm-wide test strips. 

The sample pad of an LF strip was immersed into a target sample, which migrated along the LF strip toward the absorbent pad. The MPB quantity at the test line was then found using a pre-defined calibration dependence of the MPQ signal upon the amount of MPB trapped at the test line.

### 2.5. Optical Label-Free Method of Spectral Correlation Interferometry (SCI)

The SCI method [52,53] was used in this research as a validation technique and implemented the label-free real-time registration of inter-molecular interactions. The registration was done through measuring the variations in thickness of a biomolecular layer on the sensor chip surface that occurred as a result of biochemical reactions. The method is free of parasitic contributions to the output signal due to fluctuations of the refractive index of solutions, which strongly depends on temperature. Another convenience is measurements in metrological units such as nm or pm. In this work, we used new three-channel SCI biosensors with independent supply of reagents to each channel. 

### 2.6. Immobilization of Targets on the SCI Sensor Chips

First, the microscope cover glass slip surface was carboxilated for further immobilization of biomolecules according to the protocol described in details earlier [54,55]. In brief, the microscope cover slips were thoroughly cleaned with methanol and incubated for 16 h in 3% solution of APTES in methanol. After that, 2-h incubation in 15 mM solution of succinic anhydride in dimethylformamide was implemented followed by thrice washing in methanol. The cover slips were then incubated at 105 °C for 1 h. 

Next, the carboxilated sensor chips were activated by 15-min incubation in 6.4 mg/mL solution of EDC in MES buffer with further thrice washing with PBS buffer. Then, 1 mL of 25 μg/mL of SEB or cTnI antigen in PBS buffer was deposited onto the carboxylated surface of the chip and incubated for 2 h at room temperature. After that, 1 mL of 10 mg/mL BSA in PBS buffer was added and incubated for 30 min. Then the slips were washed five times in deionized water.

### 2.7. Procedure of SCI Measuring

A solution of 20 μg/mL of free (non-immobilized) antibody against either cTnI or SEB in PBS-BSA buffer was pumped along the sensor chip surface with the immobilized target for 15 min at a flow rate of 10 μL/min in the liquid handling system of SCI biosensor followed by pumping of PBS buffer. The sensograms of target binding were recorded by the SCI-biosensors in real time as temporal dependences of the biolayer thickness on the sensor chip surface. The kinetic constants of interactions were calculated using the Langmuir model.

### 2.8. Data Processing

All the experiments were implemented at least three times. In graphs, each value represents an average, and error bars show standard deviations. Non-linear equations were solved by the generalized reduced gradient method.

## 3. Results and Discussion

### 3.1. Experimental Setup

**Figure 1:**
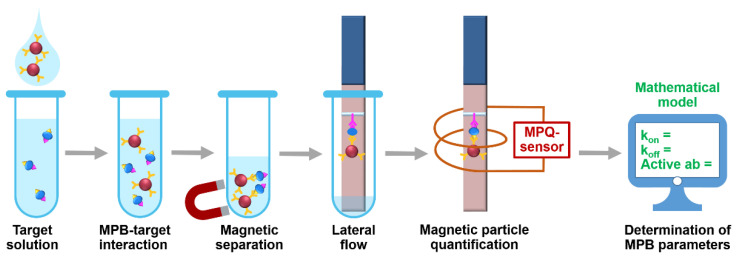
Experimental setup for rapid determining the target-binding characteristics of magnetic particle bioconjugates using the developed mathematical model.

The setup (Figure 1) realizes the following procedure. At first, MPB with immobilized biorecognition molecules, e.g., antibody, are added to a target-containing sample. During the subsequent incubation, some MPB form complexes with the target. The other MPB may remain unbound. At the second step, both bound and non-bound MPB are isolated from the sample by magnetic separation with a permanent magnet. The separated MPB are deposited onto a lateral flow strip and migrate along it due to capillary forces. Then the MPB-target complexes are entrapped at a pre-deposited test line made of, e.g., antibody to another epitope of the target. The unbound MPB do not interact with the TL and continue migrating to the absorbent pad of the LF strip. Finally, the LF strip is positioned inside a sensing coil of the MPQ sensor for accurate quantification of MPB at the TL. To determine characteristics of MPB binding with the target, we use a mathematical model described below.

### 3.2. Mathematical Model

The model is developed to represent an analytical solution of the equation of the second order bimolecular reaction:

(1) $$Mpb+Tgt_{\underset{k_{off}}{\leftarrow }}^{\overset{k_{on}}{\to }}MpbTgt,$$

where *Mpb*—magnetic particle bioconjugates, *Tgt*—targets, *MpbTgt*—MPB-target complexes. This relationship implies the following differential equation:

(2) $$\frac{d\left[MpbTgt\right]}{dt}=k_{on}\left[Mpb\right]\left[Tgt\right]-k_{off}\left[MpbTgt\right],$$

where *[Tgt]*—concentration of unbound target molecules; *[Mpb]—*concentration of MPB multiplied by fractional surface coverage; *[MpbTgt]*—concentration of MPB-bound target molecules. Here, the initial conditions are as follows:

(3) $$\;{\left[Tgt\right]}_{t=0}={\left[Tgt\right]}_{0};$$

(4) $$\;{\left[Mpb\right]}_{t=0}=\frac{m^{Mpb}}{m_{0}^{Mpb}\cdot N_{A}\cdot V};$$

(5) $$\;{\left[MpbTgt\right]}_{t=0}=0,$$

where *[Tgt]_0_*—initial concentration of target; *m^Mpb^*—mass of magnetic particle bioconjugates added to the target sample; $m_{0}^{Mpb}$—mass of a single magnetic particle bioconjugate; *N_A_*—Avogadro number; *V*—volume of the target-containing sample. Additionally, the following relationships are valid:

(6) $$\left[Tgt\right]={\left[Tgt\right]}_{0}-\left[MpbTgt\right];\;$$

(7) $$\left[Mpb\right]={\left[Mpb\right]}_{0}-\frac{\left[MpbTgt\right]}{N},$$

where *N*—initial quantity of target-binding biomolecules (TBB) per one MPB. The analytical solution of the Equations (2)–(7) results in the temporal dependence of concentration of MPB-target complexes:

(8) $$\left[MpbTgt\right]=\frac{a\cdot b\cdot \left(1-\exp\left(t\cdot k_{on}\cdot \left(a-b\right)\right)\right)}{a-b\cdot \exp\left(t\cdot k_{on}\cdot \left(a-b\right)\right)},$$

where *a* and *b* represent the expressions:

(9) $$a,\;b=\frac{{\left[Tgt\right]}_{0}+N\cdot Mpb_{0}+\frac{k_{off}}{k_{on}}}{2}\cdot \left(1\pm \sqrt{1-\frac{4\cdot {\left[Tgt\right]}_{0}\cdot N\cdot {\left[Mpb\right]}_{0}}{{\left({\left[Tgt\right]}_{0}+N\cdot {\left[Mpb\right]}_{0}+\frac{k_{off}}{k_{on}}\right)}^{2}}}\right).$$

The standard relationships between the kinetic and equilibrium constants are

(10) $${\;K}_{A}=\frac{k_{on}}{k_{off}};\;$$

(11) $${\;K}_{D}=\frac{k_{off}}{k_{on}}.$$

Remarkably, this model comprises only the parameters that either are known a priori for a particular assay (e.g., initial concentrations of reagents) or the parameters to be determined, namely, the kinetic (*k_on_* and *k_off_*) and equilibrium (*K_D_* and *K_A_*) constants of MPB interaction with target, as well as the TBB quantity per MPB (*N*). 

A wealth of models and descriptions of the second order bimolecular reactions have been reported [56,57,58]. However, to the best of our knowledge, we present here the first accurate analytical solution for determining the temporal dependence of bimolecular complexes concentration under non-zero initial concentrations of both interacting components.

### 3.3. Determining Kinetic and Equilibrium Constants of MPB-Target Interaction: Procedure and Demonstration

One of the possible ways to use the proposed method is finding the kinetic and equilibrium constants with the minimal number of rapid and easy-to-use experiments. According to the model, we need as little as three independent measurements by LF tests. These tests differ in (i) duration of the MPB-target interaction prior the LF assay and (ii) sample volume, in which the interaction occurs. After each test, MPB captured at the TL is quantified with the MPQ, and the kinetic constants are calculated using the mathematical model described in Section 3.2. The calculation represents a numerical solution of three non-linear Equation (8) containing three unknown variables: kinetic constants *k_on_* and *k_off_*, and quantity *N* of TBB per MPB. The obtained values can be further used for calculation of the equilibrium constants by Equations (10) and (11).

The method has been demonstrated by determining the kinetic and equilibrium constants of interactions for two MPB types, each recognizing its own non-sorbed target (cTnI or SEB) as models. For simplicity, we describe below the experimental procedure for any one of the targets. The procedure for the other one was similar. 

**Figure 2:**
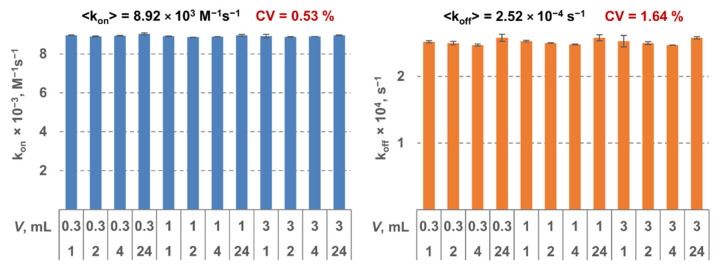
Kinetic constants k_on_ (**on the left**) and k_off_ (**on the right**) determined for staphylococcal enterotoxin B (SEB) as a model target using the proposed method at various values of sample volume and interaction duration.

The MPB was functionalized by a monoclonal antibody to the respective target. All the constants were determined using three LF tests as described above. For each MPB type, 12 triples of tests were analyzed. Two tests in each triple were done at the same conditions: 30-min interaction duration and sample volume of 0.3 mL and 10 mL, respectively. For the third test in each triple, the parameters varied: interaction durations—1 h, 2 h, 4 h, or 24 h; sample volumes—0.3 mL, 1 mL, and 3 mL. Therefore, the constants were determined using 12 independent measurements. It can be seen from Figure 2 that shows as an example the results for SEB that the obtained values are virtually independent of interaction duration and volume (the coefficient of variation <2%). That indicates high reliability of the method and its stability against the experimental conditions.

It should be noted that the membrane pore size (5–30 µm) [54] considerably exceeds MPB size and ensures quick migration of MPB along the test strip. As per manufacturer, a solution portion typically migrates through a 2-cm segment of the used membrane (from the front edge of the strip to the test line) for 50 s. According calculations with our mathematical model, at the obtained values of kinetic constants of dissociation, less than 1.5% of MPB—target complexes dissociate for that time. This fraction is very small and does not affect the results.

### 3.4. Characterization of Sorption on MPB: Total Sorbed Amount and Fraction of Active Target-Binding Biomolecules

The proposed method can be used for assessment of the total sorbed amount and the fraction of active target-binding molecules on the surface of magnetic particle bioconjugates. At first, a calibration curve is plotted as a dependence of MPB amount captured at the test line of an LF strip upon concentration of target in sample. Then an LF test is carried out with a solution of target of known initial concentration *[Tgt]_0_*, which is disenriched by 24-h incubation with MPB followed by magnetic separation. Using the calibration plot, one can find the residual concentration and calculate the concentration *[MpbTgt]* of target molecules bound by MPB.

If the dynamic equilibrium is achieved, one may use the already obtained equilibrium constants (Section 3.3) to calculate the amount of active centers with the expression:

(12) $$N\cdot {\left[Mpb\right]}_{0}=\frac{{\;K}_{D}\cdot \left[MpbTgt\right]}{{\left[Tgt\right]}_{0}-\left[MpbTgt\right]}+\left[MpbTgt\right].$$

The results of estimation of the sorption characteristics of MPB are shown in Table 1. The total amount of molecules sorbed on MPB was obtained by the BCA assay, while the portion of active target-binding biomolecules—as the ratio of the amount of active biomolecules calculated from Equation (12) to the total amount. The respective values per one particle were determined by dividing by the quantity of participating MPB. The sorption density was obtained as the number of biomolecules on a single particle divided by the surface area of the particle calculated using its radius. As can be seen from the table, the quantity of sorbed biomolecules is in the range of (25–35) × 10^3^ pieces per MPB. Meanwhile, the traditional techniques either do not allow estimating the portion of active-binding molecules or require for this purpose additional labor-intensive and costly procedures. In contrast, the proposed method provides an accurate and cost-efficient LF-based solution.

As can be seen in Table 1, the differences in size and total amount of immobilized biomolecules are very small for cTnI—MPB and SEB—MPB. At the same time, the observed amounts of active biomolecules differ almost twofold. That may be due to the effect of orientation and conformation changes, which occur during immobilization, on activity of target-binding molecules. These changes substantially depend upon properties of a particular molecule, e.g., upon post-translational modifications. In particular, it is known that various protein molecules of the same class and even the same primary structure but having different degree of glycosylation may manifest different activity, stability, and immobilization efficiency [59].

### 3.5. Validation of the Proposed Method

**Figure 3:**
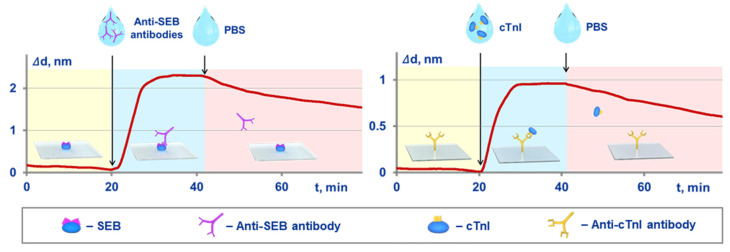
Schematic of the Spectral Correlation Interferometry (SCI)-based experiments for independent determination of kinetic and equilibrium constants of MPB-target interactions, and the related experimental sensograms recorded in real time for SEB target (**on the left**) and cTnI target (**on the right**).

An independent validation was implemented by comparison of the kinetic and equilibrium constants obtained with both the proposed method and an optical label-free method of spectral-correlation interferometry. The SCI-based experimental setup and related experimental sensograms registered in real time are shown in Figure 3. In the SCI setup, the target molecules were immobilized on the surface of glass sensor chips. The procedure for calculation of the constants was reported earlier [25,36,42]. Briefly, at first, a solution of target-binding molecules is pumped along the sensor chip surface, and the kinetic constant of association is estimated using the recorded sensogram. Upon the sensogram plateauing, a target-free solution is pumped, and the kinetic constant of dissociation is determined. The equilibrium constants are calculated using the Equations (10) and (11).

The values of kinetic and equilibrium constants obtained by both methods are compared in Table 2. Since the SCI is a label-free technique, it registers, as discussed in the Introduction, the “biorecognition molecules–target” interactions rather than the interactions of interest, which are those between the MPB functionalized by biorecognition molecules and the target. Importantly, the label-free technique can study target binding of only free (non-immobilized) molecules. In contrast, the proposed method is a tool for comprehensive characterization of magnetic nanobioconjugates, which have immobilized on their surface non-oriented biomolecules and unknown ahead quantity of target-binding sites. Therefore, for correct comparison, the MPB-target constants obtained with the proposed method were recalculated to reflect the bimolecular “antibody–target” interactions rather than the multi-point binding of MPB. Notably, the number of active molecules per particle determined earlier was required for such calculation. The similar values of the constants obtained by the substantially different methods indicate good agreement of these independent approaches.

The specificity of the presented method has been confirmed as follows. In this experimental series, a variety of non-target molecules were added to samples that contained or did not contain targets. The tested non-target molecules included human serum albumin (HSA), thyroid stimulating hormone (TSH) and prostate specific antigen as high-molecular-weight proteins; biotin and chloramphenicol as low-molecular-weight substances; a mixture of human IgG immunoglobulins. The concentrations of these substances were 10 IU/mL for TSH, 1 mg/mL for HSA, and 10 μg/mL for each other substance. The results obtained in these experiments have shown no dependence on the presence of non-target substances in the analyzed samples within the experimental error.

It is important that to describe MPB behavior, almost all label-free techniques require an additional estimation of the quantity of binding sites on MPB. In contrast, the developed method investigates MPB per se and does not need such estimations. Moreover, the method represents a unique tool for measuring the number of active target-binding centers on MPB. Hence, when it is MPB-target interaction that should be studied and characterized rather than the inter-molecular interaction with target-binding biomolecules, the proposed method is preferable to label-free techniques.

### 3.6. Quantitative Prognosis of Efficiency of Magnetic Conjugate Binding with Target

**Figure 4:**
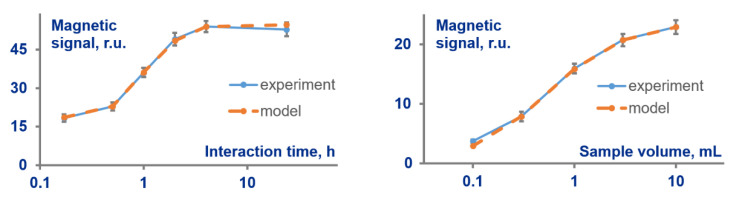
Comparison of MPB signals predicted theoretically (orange color) with the experimentally measured by the magnetic particle quantification technique quantity of MPB (blue color) trapped at the test line after interaction with target under various interaction times and target-sample volumes.

Another advantage of the developed method is its capacity to provide an accurate prognostic description of MPB interaction with target and predict its dynamics. We have demonstrated this feature by determining the number of MPB-target complexes depending on duration *t* of MPB-target interaction and sample volume *V*. To find the prognostic meaning for any arbitrary *t* and *V*, one should substitute into Equation (8) the values of kinetic constants and *N* obtained as described in Section 3.3. Figure 4 exhibits in orange color the predicted dependences of the complexes quantities upon (i) *t* in the range of 30 min—24 h, and (ii) *V* in the range (0.1–10) mL. This figure also shows in blue color the experimentally obtained, MPQ-measured values of MPB trapped at the test line after interaction with target under respective meanings of *t* and *V*. As can be seen from the figure, the theoretical and experimental values are the same within the experimental error throughout the whole wide ranges of *t* and *V*. This fact suggests exceptional reliability of the method and verifies its suitability for accurate characterization of MPB–target interactions. 

The side-by-side comparison of the developed method with the current techniques for characterization of target binding and kinetics of particle-based bioconjugates is given in Table 3.

## 4. Conclusions

The proposed method offers a tool for multi-factor accurate characterization of interaction of magnetic particle bioconjugates with non-sorbed targets. The method is based on magnetic separation, an express magnetic lateral flow quantitative assay and a mathematical model that describes formation and evolution of MPB–target complexes and does not comprise parameters to be fitted additionally.

The experimental procedure demonstrated here for cTnI and SEB as model targets needs only three user-friendly lateral flow tests to determine kinetic and equilibrium characteristics, total amount of biomolecules on the MPB surface and the fraction of which capable of active targeting. The method permits plotting a prognostic dependence of MPB targeting capacity upon a wide range of interaction durations and sample volumes. The developed method investigates MPB per se and does not need an additional estimation of the quantity of binding sites on MPB. The results were confirmed by a label-free biosensor based on the spectral-correlation interferometry.

In addition, the research presents the first accurate analytical solution for determining the temporal dependence of bimolecular complexes concentration under non-zero initial concentrations of both interacting components. The experimental demonstration and validation of the method indicate its high reliability. The method can become an efficient solution for considerable acceleration of MPB selection for a wide range of applications from development of various affinity sensors to MPB-based biosensing in vivo. 

The research promises significant opportunities for follow-up studies, e.g., assessment of the fraction of active molecules over a wide range of experimental conditions to find optimal antibody–particle ratios. Importantly, our method, which is here demonstrated for magnetic particles, can be further extended for investigation and characterization of a wide range of other submicron particles such as gold and fluorescent ones, quantum dots, etc. For this purpose, the developed mathematical model and experimental setup can be used in combination with another, e.g., optical method of particle detection.

## Figures and Tables

**Table 1:** Values of sorption characteristics of magnetic particle bioconjugates (MPBs) obtained with the proposed method.

MPB Target	MPB Diameter, nm	Total TBB Mass Per 1 g MPB, g	Active TBB Mass Per 1 g MPB, g	Total TBB Number Per 1 MPB, pcs	Active TBB Number Per 1 MPB, pcs	Total TBB Sorption Density, pcs ^1^/m^2^	Active TBB Sorption Density, pcs ^1^/m^2^
cTnI	196	1.037	0.217	3.27 × 10^4^	6.84 × 10^3^	2.71 × 10^17^	5.67 × 10^16^
SEB	213	0.781	0.121	2.61 × 10^4^	4.06 × 10^3^	2.08 × 10^17^	3.23 × 10^16^

^1^ pcs—pieces of target-binding biomolecules per one MPB; TBB—target-binding biomolecules; cTnI—cardiac troponin I.

**Table 2:** Side-by-side comparison of values for constants obtained with the presented method and an independent optical label-free technique of spectral-correlation interferometry

Method	Target	Interaction	k_on_, M^−1^s^−1^	k_off_, s^−1^	K_A_, M^−1^	K_D_, M
Label-free SCI	cTnI	TBB–target	(7.9 ± 0.8) × 10^4^	(2.5 ± 0.4) × 10^−4^	(3.1 ± 0.6) × 10^8^	(3.2 ± 0.6) × 10^−9^
Proposed here	cTnI	TBB–target	(7.3 ± 1.0) × 10^4^	(2.9 ± 0.2) × 10^−4^	(2.5 ± 0.4) × 10^8^	(4.0 ± 0.6) × 10^−9^
Proposed here	cTnI	MPB–target	(6.8 ± 1.4) × 10^7^	(2.8 ± 1.2) × 10^−7^	(2.4 ± 1.2) × 10^14^	(4.1 ± 2.0) × 10^−15^
Label-free SCI	SEB	TBB–target	(1.5 ± 0.3) × 10^4^	(3.3 ± 1.0) × 10^−4^	(4.6 ± 1.7) × 10^7^	(2.2 ± 0.8) × 10^−8^
Proposed here	SEB	TBB–target	(8.4 ± 0.9) × 10^3^	(2.4 ± 0.3) × 10^−4^	(3.4 ± 0.6) × 10^7^	(2.9 ± 0.5) × 10^−8^
Proposed here	SEB	MPB–target	(1.1 ± 0.3) × 10^7^	(3.0 ± 1.7) × 10^−7^	(3.5 ± 2.2) × 10^13^	(2.8 ± 1.7) × 10^−14^

**Table 3:** Comparison of current methods for characterization of target binding and kinetics of bioconjugates

Method	Time	Instrument	Easy-To-Use	Amount of Total Ab	Amount of Active Ab	Kinetics of TBB	Kinetics of MPB	Equilibrium Constants	Prognostic Ability	Ref.
Proposed method	20 min	Magnetic detector	YES	YES	YES	YES	YES	YES	YES	Present work
Lateral flow methods	5–30 min	Magnetic or optical detector	YES	NO	NO	NO	YES	NO	NO	[60]
Label-free techniques	30 min—4 h	Label-free detector	NO	NO	YES	NO	YES	YES	NO	[42,61]
Radioactive labels-based methods	Up to 12 h	Detector of radiolabels	NO	YES	YES	NO	YES	YES	NO	[62,63]
Optomagnetic combined with supernatant assay	Up to 6 h	Optomagnetic biosensor platform	NO	YES	YES	YES	YES	YES	NO	[64]
Mathematical model-based methods	Depends on experiments required	Depends on experiments required	NO	NO	YES	YES	YES	YES	YES	[65]
ELISA-based methods	2–16 h	Microplate and plate reader	NO	YES	YES	NO	NO	YES	NO	[66,67,68]
SDS-PAGE analysis	2 h	Phast system apparatus	NO	YES	NO	NO	NO	NO	NO	[67,69]
Fluorescence-based assays	1–2 h	Spectrofluorimeter	NO	YES	NO	NO	NO	NO	NO	[70,71,72]
BCA/Bradford/ Lowry assays	30–60 min	Microplate and plate reader	YES	YES	NO	NO	NO	NO	NO	[68,73,74]
